# Perspectives and Experiences of Family Caregivers Using Supportive Mobile Apps in Dementia Care: Meta-Synthesis of Qualitative Research

**DOI:** 10.2196/65983

**Published:** 2025-06-18

**Authors:** Haifei Shen, Yi Han, Wen Shi, Jiangxuan Yu, Xueqi Shan, Hongyao Wang, Junjie Wang

**Affiliations:** 1 School of Nursing Zhejiang Chinese Medical University Hangzhou China

**Keywords:** dementia, caregivers, mobile app, qualitative research, meta-synthesis, artificial intelligence, AI

## Abstract

**Background:**

Supportive mobile apps are effective tools for family caregivers of persons with dementia to obtain online information and psychological support. Nevertheless, details about the experiences of family caregivers of persons with dementia using mobile apps are limited.

**Objective:**

This study aimed to synthesize the perspectives and experiences of family caregivers of persons with dementia regarding supportive mobile apps.

**Methods:**

We conducted a synthesis of qualitative research and searched 7 English-language databases and 4 Chinese-language databases. We included qualitative studies (peer-reviewed studies and gray literature) written in English and Chinese on the perspectives and experiences of family caregivers of persons with dementia regarding supportive mobile apps published from database establishment to March 2025. Two researchers independently screened the literature and used the JBI Critical Appraisal Checklist for Qualitative Research to conduct quality assessments on the final included studies. Themes were integrated using the 3-stage thematic synthesis approach by Thomas and Harden.

**Results:**

A preliminary search yielded 4772 studies, of which 12 (0.25%) met the criteria. The included studies were from 7 different countries or regions, of which the only low- or middle-income country was Brazil. The studies involved a total of 232 family caregivers, most of whom were older adults and female. The integration of extracted content resulted in 4 themes: dynamic changes in value perception—complex attitudes toward mobile app adoption; from tools to partners—a technology-empowered multidimensional support system for family caregivers; external and internal barriers—challenges in family caregivers’ use of mobile apps; and person-centered design—future directions for improving mobile apps.

**Conclusions:**

This study found that family caregivers’ attitudes toward using supportive mobile apps are influenced by their perceived value of mobile apps and their caregiving burden. In addition, such supportive mobile apps serve as valuable tools for family caregivers to enhance their caregiving abilities and efficiency, alleviate the burden of care, improve negative emotions, foster social connections, and promote self-care. Future mobile app design needs to address obstacles such as design flaws, family caregivers’ lack of technological literacy, time constraints, concerns about privacy breaches, and other device-related issues, with particular attention to the ease of use of mobile apps. Meanwhile, developers need to commit to designing personalized and multifunctional mobile apps as well as promote online collaboration among members of the care network. Overall, our study offers an important reference for developing person-centered supportive mobile apps for family caregivers of persons with dementia.

**Trial Registration:**

PROSPERO CRD42024510905; https://www.crd.york.ac.uk/PROSPERO/view/CRD42024510905

## Introduction

### Background

The phenomenon of an aging global population has significantly impacted the prevalence of dementia [1]. According to the World Alzheimer Report (2023), the global population of persons with dementia is anticipated to increase from 55 million in 2019 to 139 million by 2050 [2]. Dementia is a chronic, long-term, progressive disease [3]. As dementia progresses, persons with dementia gradually become incapable of caring for themselves and develop cognitive and behavioral abnormalities, requiring substantial assistance from caregivers for many daily activities in the later stages [3]. The World Health Organization declared dementia a public health priority and committed to the care of persons with dementia and the assistance of their carers in 2017 [4]. Persons with dementia are mainly taken care of by family members, which includes spouses, children, other relatives, and friends [5]. Family caregivers of persons with dementia face higher physical and psychological challenges than typical caregivers because of their long-term exposure to various stresses [6], which indirectly reduces the quality of care [7] and can lead to social issues such as intrafamily conflicts, patient abuse, and even caregiver suicide [8]. At the same time, family caregivers lack knowledge and skills related to the progression and management of dementia [9], leading to low confidence in caregiving. Inappropriate care behavior could even trigger behavioral and psychological symptoms of dementia in persons with dementia [10]. Therefore, family caregivers urgently need knowledge, skills, and emotional support.

Acceptance and commitment therapy, cognitive therapy, psychoeducation, and other interventions could effectively alleviate family caregivers’ stress and provide support for them [11]. However, most existing interventions are face-to-face, which can be labor intensive and time-consuming and only help a restricted number of people. Furthermore, due to limited health care and caregiving resources [12], there is an urgent need for innovative alternative approaches and methods to promote convenient and efficient dementia care and self-management for family caregivers.

Supportive measures based on mobile health (mHealth) may be an effective alternative. The World Health Organization defined mHealth as “medical and public health practice supported by mobile devices, such as mobile phones, patient monitoring devices, PDAs, and other wireless devices” [13]. Mobile apps are one of the main forms of mHealth and generally refer to app software downloaded on smart mobile terminals such as smartphones [14]. These mobile apps, increasingly used to support family caregivers of persons with dementia, accounted for 18 out of 26 (69%) chronic illness caregiver apps in a review, highlighting their potential [15]. In our study, supportive mobile apps can be defined as a type of mobile app designed for family caregivers of persons with dementia whose functions include knowledge transfer, assistance with caregiving tasks, and provision of social and psychological support, aiming to help reduce caregiving stress and negative emotions in family caregivers as well as improve the quality of caregiving [16-18]. Compared to traditional interventions, mobile apps overcome time and space limitations, enabling around-the-clock access to resources, which is especially beneficial for remote users seeking services [19]. In contrast to other web-based applications, they allow for offline use, access to built-in components, or connection to external devices [15]. These mobile apps are critical for family caregivers, especially when dealing with the most challenging behavioral and psychological symptoms of dementia of persons with dementia. They not only provide care knowledge and social service information, reducing information retrieval burdens [18], but also include monitoring functions to help assess the status of persons with dementia. In addition, around-the-clock access and online communication ensure that family caregivers can obtain timely advice for managing challenges [20]. Studies have shown that mobile apps significantly alleviate family caregivers’ caregiving burden and depressive symptoms [21,22] while enhancing their caregiving competence and quality of life [23]. With technological advancements, this support has increasingly become more precise. For instance, the Olera.care platform constructs user profiles through needs questionnaires and algorithm models to achieve precise content delivery and educational resources [24]. Lukkien et al [25] developed a dementia care knowledge base using natural language processing technology and implemented intelligent question-and-answer support through chatbots. However, most existing technological innovations remain limited to web-based applications, with mobile app implementations and related research still insufficient. Future studies should explore mobile implementations to enhance support for family caregivers of persons with dementia.

Although mobile apps demonstrate considerable potential in supporting family caregivers, research findings remain inconsistent. Several randomized controlled trials and quasi-experimental studies have shown that, compared to control groups, family caregivers using supportive mobile apps exhibit no significant improvements in caregiving burden [26], caregiving competence [21], or depressive symptoms [26]. A study by Rodriguez et al [20] revealed a decline in family caregivers’ intention to use a mobile app (Brain CareNotes) after the 6-month follow-up, indicating challenges in sustaining long-term engagement. Additional studies highlight further obstacles, including low user satisfaction, underuse of specific functions [21], and concerns regarding security and privacy [27]. An evaluation of mobile apps for persons with dementia and their family caregivers also revealed quality deficiencies and insufficient functionality design to meet family caregivers’ actual needs effectively [28]. These inconsistent results all indicate the need to fully consider family caregivers’ complex experiences, individualized needs, and specific caregiving contexts in the design of mobile apps. Relying solely on quantitative indicators may be insufficient to fully explain the reasons behind these inconsistencies.

### Objectives

Qualitative methods uniquely uncover user experiences, explain anomalies in quantitative results, and identify overlooked issues [29]. Thus, capturing users’ emotional feedback and experiences is vital for optimizing mobile apps. While qualitative studies examine family caregivers’ perceptions of mobile apps, individual studies cannot capture the entirety of their experiences. For instance, Chapman et al [30] showed that the community function strongly supports family caregivers, whereas Dorell et al [31] found that not all users prefer group interactions. Moreover, existing systematic reviews mainly focus on quantitative research evidence to explore intervention effects on family caregivers [23,32,33]. Although a few reviews have included qualitative studies, the number of these studies was limited, primarily focusing on assessing mobile apps’ functionality [18]. Another systematic review provided a comprehensive analysis of the features, security, and usability of mobile apps in dementia care [27]. However, the reviewed mobile apps predominantly focused on supporting persons with dementia. There is still a need for further exploration of the support that family caregivers receive from mobile apps [27]. Meta-synthesis can integrate multiple perspectives to analyze problems, resulting in a more comprehensive and in-depth understanding of complex phenomena [34]. No previous study has synthesized qualitative research on family caregivers’ experiences, preferences, and challenges regarding supportive mobile apps*.* Given the growing mHealth reliance in caregiving, addressing this gap is urgent. Consequently, this study aimed to conduct a meta-synthesis of existing qualitative research to deeply analyze common experiences and individual differences in family caregivers’ use of supportive mobile apps. The results will facilitate the creation and improvement of future relevant mobile apps that are grounded in person-centered principles to alleviate the psychological and physical stress of family caregivers and enhance the quality of care.

## Methods

### Study Design

This meta-synthesis adhered to the ENTREQ (Enhancing Transparency in Reporting the Synthesis of Qualitative Research) [35] and PRISMA (Preferred Reporting Items for Systematic Reviews and Meta-Analyses) [36] guidelines. The specific details can be found in Multimedia Appendix 1. The research protocol has been registered in PROSPERO, with registration CRD42024510905.

### Search Strategy

A total of 7 English-language databases (the Cochrane Library, Embase, CINAHL, PubMed, PsycINFO, Scopus, and Web of Science Core Collection) and 4 Chinese-language databases (China National Knowledge Infrastructure, Wanfang Data, Weipu, and SinoMed) were searched from database establishment to March 2025. First, primary keywords were established based on existing knowledge, and then preliminary searches were conducted in the English-language database PubMed and the Chinese-language database China National Knowledge Infrastructure. We carefully reviewed the titles, abstracts, and keywords of relevant papers to establish comprehensive and specific search strategies tailored to each database. Second, we searched each database according to its search strategy. Third, references to the literature [30,31,37-46] were reviewed to identify additional studies. The specific search strategies are provided in Multimedia Appendix 2.

### Eligibility Criteria and Study Selection

Studies were screened according to the inclusion and exclusion criteria shown in Textbox 1. In total, 2 researchers (HS and YH), who were trained in evidence-based methodology, independently screened the studies, extracted data, and cross-checked their work. If there were any disagreements, they instantly consulted a third researcher (WS) for evaluation. First, the search results were imported into EndNote (version 21; Clarivate Analytics), and duplicate references were deleted. Second, a preliminary screening of studies was conducted by reading the titles and abstracts to identify works for further scrutiny. Finally, a full-text review of the studies selected was conducted to confirm their final inclusion in the review.

**Inclusion and exclusion criteria.**

**Inclusion criteria**

- Population: family caregivers of persons with dementia, such as spouses and offspring (if the study population included both persons with dementia and their family caregivers, only studies involving mobile apps with explicitly defined functions dedicated solely to family caregivers were included)
- Phenomena of interest: studies that explored the attitudes, expectations, or experiences of family caregivers regarding the use of supportive mobile apps (the mobile apps must explicitly include at least one function directly related to caregiving support)
- Context: family caregivers use supportive mobile apps in any environment, including in the home, community, hospital office, and others
- Study design and publication type: all types of qualitative studies and the qualitative components of mixed methods research

**Exclusion criteria**

- Population: formal caregivers (eg, nurses)
- Phenomena of interest: digital tools such as websites, telephone services, and SMS text messaging services and no specific functions in the mobile apps designed to support family caregivers of persons with dementia
- Study design and publication type: non–English- and non–Chinese-language papers, conference papers, and reviews; studies with no qualitative content or for which we were unable to extract valid qualitative content; and no access to the full text

### Appraisal of Methodological Quality

In total, 2 researchers (HS and YH) independently assessed the quality of the studies based on the JBI Critical Appraisal Checklist for Qualitative Research [47]. The assessment consists of 10 items, which can be rated as “yes,” “no,” “unclear,” or “not applicable.” Disagreements were resolved by involving a third person (WS).

### Data Extraction

The data extracted included the author, publication year, country or region, research method, data collection method, study population, phenomena of interest, main findings, participant quotations, and relevant author interpretations in the papers. Two researchers (HS and YH) independently extracted the data; a third researcher (WS) checked the data. Participant quotations and relevant author interpretations from the papers were further entered into Microsoft Excel (Microsoft Corp) for coding.

### Data Synthesis

This study applied the thematic synthesis method proposed by Thomas and Harden [48] to the qualitative synthesis. This process has three stages: (1) coding text, (2) organizing codes into descriptive themes, and (3) generating analytical themes. Initially, 2 researchers (HS and YH) conducted line-by-line coding of textual data concerning family caregivers’ perceptions of and experiences with mobile apps. We systematically compared qualitative descriptions across the studies to identify commonalities and variations, ensuring that the generated codes both captured core research content and preserved original contextual meanings. Discrepancies in coding were resolved through discussion with a third researcher (WS) until consensus was reached. Before completing this stage, we also reviewed all texts associated with the codes to ensure consistency in interpretation. Following this, researcher 1 (HS) continuously compared the differences and logical relationships among the codes to inductively and iteratively generate descriptive themes, which were then evaluated and finalized by 2 team members (YH and WS). In the final stage, researcher 1 (HS) designated analytical themes based on descriptive themes to transcend the findings of individual studies and produce a higher level of conceptual understanding. The broader team subsequently reviewed this process to ensure its rigor. Microsoft Excel was used for data organization.

Reciprocal translation and constant comparison permeated the thematic synthesis. For instance, in study 1, a family caregiver saying that they “perceived the app STAV as a source of stability that helped her to stay afloat in her daily life as a caregiver” was translated as “the app provides stability in daily lives.” A participant in study 2 stated, “Yes, but it felt good with this app, I did not use it much, but it was there somehow then, and it felt good to have it...” which was translated as “the existence of the app brings positive feelings.” In study 3, a family caregiver mentioned, “I feel that I have my guardian angel, my companion, my security guard. We feel more relaxed and safer,” which was translated as “the app creates a sense of security and companionship.” Although there were some differences in the translation results of these 3 studies, the concepts of “stability, positive feelings, security, and companionship” all pointed toward the positive impact of mobile apps on family caregivers’ psychological well-being, with a core focus on their role in providing psychological stability. Considering the overall context, these subtle differences did not affect the consistency of the overall concept. Therefore, we unified these 3 statements under the code “mobile app as a source of psychological stability.”

## Results

### Search Results

**Figure 1:**
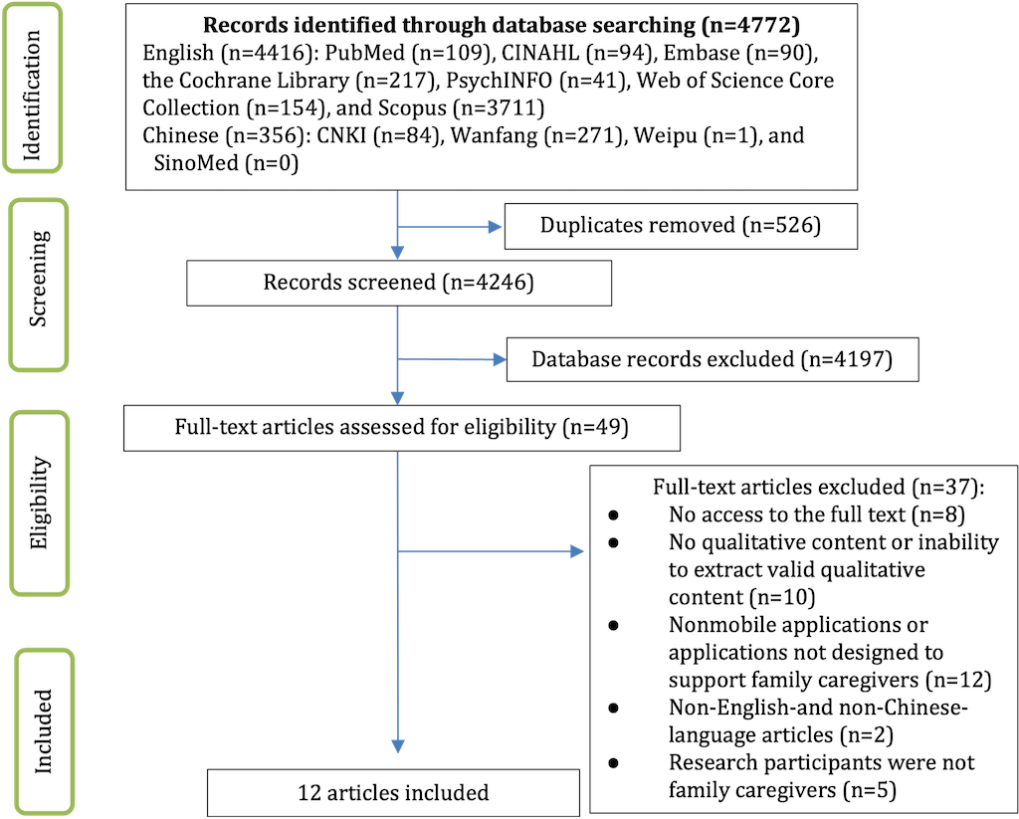
PRISMA flow diagram showing the study selection process. CNKI: China National Knowledge Infrastructure.

A comprehensive search of published qualitative research yielded 4772 studies. After the removal of duplicates and an examination of the titles and abstracts, of the 4772 studies, 49 (1.03%) were selected for full-text review. A total of 12 (0.25%) studies that met the requirements were ultimately included. Upon reviewing the references of the 12 studies, no more relevant studies were included. Figure 1 shows the screening procedure.

### Quality Appraisal

Table 1 shows the quality assessment of the 12 included studies. None of the studies fully met the standards of the Joanna Briggs Institute, but 17% (2/12) studies met 9 items, 17% (2/12) studies met 8 items, 58% (7/12) studies met 7 items, and 8% (1/12) study met 6 items. The problematic items were that only 25% (3/12) reported the methodology, 33% (4/12) reported the researchers’ circumstances, and none reported the researchers’ influence on the research.

**Table 1:** Quality assessment of the included studies based on the JBI Critical Appraisal Tool.

Study	JBI Critical Appraisal Tool item									
	1^a^	2^b^	3^c^	4^d^	5^e^	6^f^	7^g^	8^h^	9^i^	10^j^
Hussein et al [37], 2024	Yes	Yes	Yes	Yes	Yes	No	No	Yes	Yes	Yes
Kagwa et al [38], 2024	Unclear	Yes	Yes	Yes	Yes	No	No	Yes	Yes	Yes
Llaneza et al [39], 2024	Yes	Yes	Yes	Yes	Yes	Yes	No	Yes	Yes	Yes
Castillo et al [40], 2024	Unclear	Yes	Yes	Yes	Yes	No	No	Yes	Yes	Yes
Chapman et al [30], 2023	Unclear	Yes	Yes	Yes	Yes	No	No	Yes	Yes	Yes
Mishra et al [41], 2023	Unclear	Yes	Yes	Unclear	Yes	No	No	Yes	Yes	Yes
Dorell et al [31], 2022	Unclear	Yes	Yes	Yes	Yes	No	No	Yes	Yes	Yes
Kagwa et al [42], 2022	Yes	Yes	Yes	Yes	Yes	Yes	No	Yes	Yes	Yes
Leung et al [43], 2022	Unclear	Yes	Yes	Yes	Yes	Yes	No	Yes	Yes	Yes
Désormeaux-Moreau et al [44], 2021	Unclear	Yes	Yes	Yes	Yes	Yes	No	Unclear	Yes	Yes
Brites et al [45], 2020	Unclear	Yes	Yes	Yes	Yes	No	No	Yes	Yes	Yes
Ruggiano et al [46], 2019	Unclear	Yes	Yes	Yes	Yes	No	No	Yes	Yes	Yes

^a^Is there congruity between the stated philosophical perspective and the research methodology?

^b^Is there congruity between the research methodology and the research question or objectives?

^c^Is there congruity between the research methodology and the methods used to collect the data?

^d^Is there congruity between the research methodology and the representation and analysis of the data?

^e^Is there congruity between the research methodology and the interpretation of the results?

^f^Is there a statement locating the researcher culturally or theoretically?

^g^Is the influence of the researcher on the research, and vice versa, addressed?

^h^Are participants, and their voices, adequately represented?

^i^Is the research ethical according to current criteria or, for recent studies, and is there evidence of ethics approval by an appropriate body?

^j^Do the conclusions drawn in the research report flow from the analysis or interpretation of the data?

### Characteristics of the Included Studies

Table 2 reports the characteristics of the qualitative components of all studies. The studies were conducted in 7 countries or regions, namely, the United States (4/12, 33%); Sweden (3/12, 25%); the United Kingdom (1/12, 8%); Australia (1/12, 8%); Brazil (1/12, 8%); Canada (1/12, 8%); and Hong Kong, China (1/12, 8%). A total of 67% (8/12) of the studies reported the types of mobile apps and their long-term use by family caregivers. In total, 33% (4/12) of the studies involved family caregivers who had only learned about and experienced mobile apps on the day of the interview or a few days before. A total of 25% (3/12) of the studies were qualitative reports on the same mobile app. The functions involved assessment, information and external resources, tracking and monitoring, communication platforms, reminders, stress management, care coordination, and digital painting. A total of 232 family caregivers were included. In total, 17% (2/12) of the studies, which were mixed methods studies, did not report the demographic characteristics of the participants. On the basis of the available data, the remaining studies primarily involved female participants (n=115), spouses (n=62), and offspring (n=52). A total of 33% (4/12) of the studies used mixed methods, and the rest (8/12, 67%) were qualitative studies. The methods included semistructured interviews (6/12, 50%), focus groups (5/12, 42%), and open-ended questions (1/12, 8%).

**Table 2:** Characteristics of the included studies.

Study	Study design and method of data collection and analysis	Participants	Phenomenon of interest	Type of app and use	Main findings (themes)
Hussein et al [37], 2024, Australia	Exploratory descriptive qualitative study; focus group; inductive thematic analysis	N=6 (female: 83%; spouses: 33%; offspring: 33%); average age 61.3 (range 30-85) y	To identify potential barriers to and facilitators of the implementation of PainChek by family caregivers of persons with dementia	PainChek helps caregivers generate pain assessments for persons with dementia through facial expressions or nonfacial input methods; presented with a brief video on PainChek and how to use it during the focus group	Three themes and 13 subthemes: (1) user-related factors (age of attending physician and family carer, physical limitations, skills and knowledge of mHealth^a^ apps, previous experience with mHealth apps, family caregivers’ knowledge of pain in dementia, and willingness to use PainChek), (2) intervention-related factors (PainChek; cost, technical issues, misinterpretations of how PainChek works, and consequences of PainChek use), and (3) contextual factors (collaboration and trust; health care team’s acceptance, influence of family, pain communication, documentation, and discussion)
Kagwa et al [38], 2024, Sweden	Qualitative descriptive study; semistructured interviews; thematic analysis	Community-based social care professionals: n=11; family caregivers: n=19 (female: 84%; spouses: 68%; offspring: 16%); age ranging from 53 to 85 y	To explore how social care professionals and family caregivers of persons with dementia living at home experience providing and receiving support through a tailor-made mobile app named STAV^b^	STAV functions include chat, mindfulness exercises, web links to relevant sites, own contact list, and personal diary; use of the app for 8 wk	Three themes and 9 subthemes: (1) accessibility to support—bridging the gap (offering flexibility and enabling privacy), (2) engaging from a distance (varied levels of engagement, engagement with technology, navigating support, and forming a connection), and (3) limitations of the support (time lag in communication, limited functions in complex situations, and necessity of digital literacy)
Llaneza et al [39], 2024, United States	Qualitative descriptive study; semistructured interviews; inductive and deductive approaches	N=15 (female: 93%; spouses: 33%; offspring: 53%); average age 61.86 (range 34-80) y	To explore the experiences of family caregivers of older adults with cognitive impairment using a mindfulness therapy mobile app, determining facilitators and barriers	Mindfulness coach app; use of the app for 8 wk	Six themes: (1) convenience (flexibility to fit app use into individual schedules), (2) barriers (life and caregiving responsibilities interfere with daily app use), (3) perceived helpfulness (mindfulness coach as a source of knowledge and skill), (4) useful features (unique aspects of the app that increased mindfulness training), (5) suggested app improvements (future directions of app development for caregivers), and (6) mindfulness transfer (use of mindfulness skills in daily routines without opening the app)
Castillo et al [40], 2024, United Kingdom	Mixed methods study; semistructured interviews; thematic analysis	N=35; demographic characteristics not reported	To explore the perceptions and unmet needs of family caregivers of persons with dementia regarding the use of supportive apps	App 1: Dementia Talk—provides information about dementia, tracks observed behaviors, and offers stress-related management; app 2: CLEAR^c^ Dementia Care app provides information about dementia and allows to record observed behaviors; use of the app for 2 wk	Four themes and 15 subthemes: (1) positive aspects (informative, visual appeal and layout, and facilitated monitoring of behaviors and symptoms), (2) negative aspects (difficulty navigating the app, lack of customization, no added value, and presentation of the information), (3) content and feature considerations (connections to other supports, record keeping and monitoring, information and tips, strategies to manage stress, and coordination of care), and (4) user experience considerations (ease of use, layout, and cost)
Chapman et al [30], 2023, United States	Mixed methods study; in-depth, semistructured interviews; a priori qualitative data coding system to extract relevant interview themes	N=10 (female: 40%; spouses: 80%; offspring: 20%); average age 66.2 y	To gauge the usability of a new, multicomponent (mHealth) tablet app for family caregivers of persons with dementia	The CARE^d^-well app functions include assessments, psychoeducation, community, managing care, video hub, and goals; use of the app at least 4 times per wk for 1 mo	Three themes: (1) technical, (2) content and specific sections feedback, and (3) general feedback
Mishra et al [41], 2023, United States	Mixed methods study; open-ended questions; videoconferencing or in-person meetings; inductive approach	N=14 (female: 85%); average age 68 y	To explore the perceptions of family caregivers of persons with dementia regarding the acceptability and feasibility of the care coordination platform Care4AD	Care4AD app functions include coordinating care, monitoring physical activity, and scheduling reminders; demonstration of the mobile app to family caregivers before filling out the open-ended questionnaire	Five themes: (1) perceived ease of use, (2) perceived usefulness, (3) attitude toward use, (4) concerns about data privacy and sharing, and (5) technology anxiety
Dorell et al [31], 2022, Sweden	Qualitative descriptive study; semistructured interviews; thematic analysis	N=12 (female: 58%; spouses: 92%; offspring: 8%); age was not reported	To describe the experiences of family caregivers of persons with dementia who received professional support through a mobile app and its use	STAV functions include chat, mindfulness exercises, web links to relevant sites, own contact list, and personal diary; use of the app for 4 to 16 wk	Six themes: (1) filling a gap, (2) right time and right place, (3) foundation for inner calm, (4) better introduction and overcoming technical barriers, (5) relevant information in one place, and (6) way of offloading
Kagwa et al [42], 2022, Sweden	Qualitative explorative study; semistructured interviews; content analysis	N=12 (female: 58%; spouses: 92%; offspring: 8%); age was not reported	To explore how family caregivers of persons with dementia living at home, as consumers of health care services, cocreate value in a multi-stakeholder context through a tailor-made mHealth app	STAV; the functions include chat, mindfulness exercises, web links to relevant sites, own contact list, and personal diary; use of the app for 8 wk	Eight themes: (1) cooperating, (2) collating information, (3) combining complementary therapies, (4) colearning, (5) changing ways of doing things, (6) connecting, (7) coproduction, and (8) cerebral activities
Leung et al [43], 2022, Hong Kong, China	Mixed methods; semistructured focus group interview; content analysis	N=14; demographic characteristics not reported	To assess the feasibility and acceptability of having family caregivers of persons with dementia draw electronic paintings using a mobile app and assess the preliminary effect of the intervention on their well-being	e-Painting app functions included picture sharing, painting, chat room, announcements, and self-assessment; use of the app for 8 wk	Five themes: (1) preferences with regard to features and expected training, (2) satisfaction and enjoyment in the use of the e-painting app, (3) the app as a channel to vent emotions, (4) the app made them feel connected, and (5) combating the challenges of caregiving
Désormeaux-Moreau et al [44], 2021, Canada	Qualitative study; focus group interview; content analysis	N=4 (female: 100%; spouses: 50%; offspring: 50%); average age 68.25 (range 58-78) y	To explore whether family caregivers view mobile apps as relevant to meeting their needs and as useful in managing disruptive behaviors and document the types of mobile apps that are of interest and appeal to most caregivers	8 apps; these apps’ functions included providing dementia-related information and strategies for managing disruptive behaviors, reminders, live assistance, sharing, and scenario simulation; familiarization with the apps for 1 wk before the focus group	Two themes: (1) relevance of the mobile apps and (2) perceived usefulness of the mobile apps
Brites et al [45], 2020, Brazil	Qualitative study; 3 focus groups; thematic analysis	N=20 (female: 90%; spouses: 15%; offspring: 75%); average age 67.00 y	To analyze the perceptions of family caregivers and health professionals of a mobile app used for the caring and social support of persons with dementia	SMAI^e^ functions included medication reminders, alarms, the ability to send reports and images, a GPS, and a chat with medical staff; 3 focus groups were organized after 6, 12, and 18 months of app use	Nine themes: (1) communication, (2) medication management, (3) feelings of caregivers, (4) impact of dementia on the lives of caregivers, (5) caregivers’ illness, (6) care strategies, (7) caregiver safety, (8) communication with the health team, and (9) changes in daily care
Ruggiano et al [46], 2019, United States	Qualitative study; focus group interview; thematic analysis	N=36 (female: 72%; spouses: 28%; offspring:44%); average age 65.7 (range 42-89) y	To learn more about family caregivers’ perspectives on how technology can improve dementia care through beta-test interviews for a family caregiver app	Care IT functions included education, tracking and monitoring, self-assessment, and linking of caregivers to dementia resources; demonstration of the functionality of the app to family caregivers before the interview began	Three themes and 3 subthemes: (1) current technology use, (2) usefulness of technology (linking members of the care network, facilitating meaningful interactions, and personalized technologies), and (3) ease of use

^a^mHealth: mobile health.

^b^STAV: STöd till AnhörigVårdare: Support to family caregivers.

^c^CLEAR: Congnition; Life Story & Personality; Emotional & Physical Wellbeing; Activity Environment, and Relationships.

^d^CARE: Caregiver Assessment, Resources, and Education.

^e^SMAI: Sistema Móvel de Assistência ao Idoso: Mobile Care System for Older Adults.

### Main Findings of the Meta-Synthesis

#### Overview

**Figure 2:**
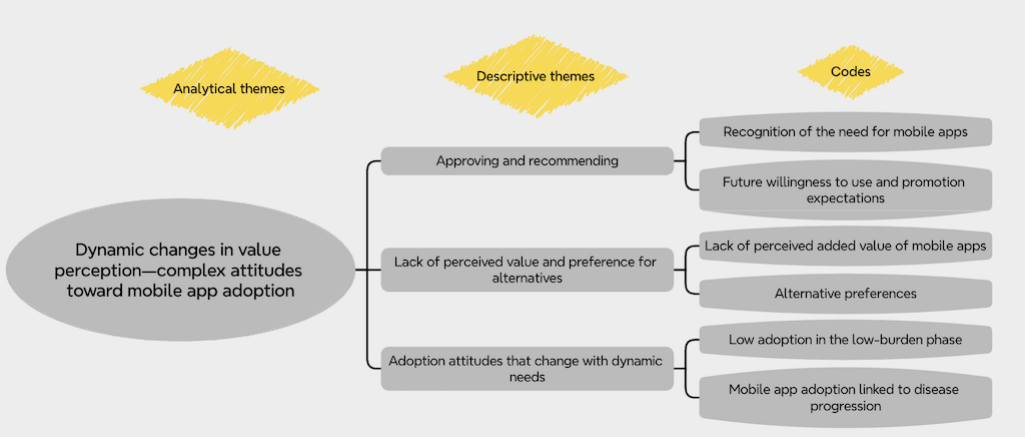
Part of the thematic synthesis process.

The thematic synthesis ultimately yielded 38 codes, 12 descriptive themes, and 4 analytical themes. Figure 2 illustrates part of the thematic synthesis process, and the specific details of the synthesis can be found in Multimedia Appendix 3. Table 3 shows the specific themes and supporting quotes.

**Table 3:** Specific themes and supporting quotes.

Analytical themes and descriptive themes		Quotes
**Dynamic changes in value perception—complex attitudes toward mobile app adoption**		
	Approving and recommending	“I think the idea of it [app] is brilliant. There’s definitely a need among caregivers...I was searching for something like this, and I just couldn’t find it before this.” [30] “The family caregivers also said that they would recommend the app to others if it was available...Most caregivers had a positive response to the mobile app.” [31]
	Lack of perceived value and preference for alternatives	“An app may not be a sufficient format to deliver resources: ‘I would have rather had a book with that information than an app to read about strategies and on how to deal with some behavioral issues.’” [40] “Some felt like the mindfulness feature was not suitable for them or achieved relaxation through other activities...I sit by the sewing machine and there is my mindfulness. There I can relax.” [38]
	Adoption attitudes that change with dynamic needs	“It was too early in his [the person with dementia] disease to get such an access [the app], really...it would be more beneficial at a later stage of dementia when the condition had deteriorated.” [42]
**From tools to partners—a technology-empowered multidimensional support system for family caregivers**		
	Achieved a convenient and efficient user experience	“Once I got to the actual program itself, it’s easy to use. I mean, it’s very clear...I found it pretty easy to maneuver, you know, to get to the different areas.” [30] “Wherever you are, if you have it on your phone, you can just stop and you know, actually gather your thoughts, no matter what the situation is.” [39] “We were unable to resolve things with her doctor, because the appointment was scheduled for two or three months in the future. I sent messages and photos, then received the response, within the timescale.” [45]
	Facilitated the optimization of care processes	“Participants reported that the information presented in the app was comprehensive and would be helpful...I just found it very supportive and informative because I’ve had no experience on this.” [40] “It was good to have the notes so as not to forget points of discussion. It was also used as a timeline for the progress of the disease as it is difficult to notice small changes in a person with dementia when together all the time.” [31] “I used to pay more attention to coughing and urination, now I pay attention to the smell of her urine. One day my husband asked me: do you smell my mother’s urine? I said: yes, of course, we need to see if it smells strong, because it could be a urinary tract infection. These two observations were excellent for me.” [45]
	Increased social connections and caregivers felt supported	“Great support, I felt a support, from the health care, from you. When I was home alone with the problems, I had to ask and could get answers pretty quickly.” [31] “The community section of the application probably turned out to be the most substantial for me, in that there were other caregivers sharing their insight and ideas about their life with their loved ones, and the issues they were having.” [30]
	Regulated negative emotions	“To focus in on my breathing really helped to reduce stress. It helped to kind of eliminate any extraneous background noises or background thoughts.” [39] “My emotions have become stable these days...Those paintings...make a person more positive, and make the emotions better.” [43]
	Brought intrinsic changes in self-care	“I feel that I have my guardian angel, my companion, my security guard. The difference is the security. We feel more relaxed and safe.” [45] “The family caregiver further expressed that it had taken her a while to realize that she could use STAV for her personal needs instead of solely focusing on the needs of the PWD whom she cared for.” [38] “I haven’t been using it recently because I sort of absorbed the habit into my life in a way, a way that I didn’t necessarily need the app...the app sort of was like a starting point or a jump-off point for me to incorporate more of that part of thinking and action in my life.” [39]
**External and internal barriers—challenges in family caregivers’ use of mobile apps**		
	External environment and technology factors	“I couldn’t really stop what I was doing and go do the app.” [39] “Participants were less likely to use mobile apps that required more steps to find needed information...one participant did not like Care4Dementia because there was ‘too much research.’” [44] “However, some caregivers experienced that they did not get answers from the nurse in time or it was too sporadic, so they stopped using the chat.” [31]
	Intrinsic abilities or psychological factors	“Both the social care professionals and the family caregivers expressed experiencing challenges for the family caregivers with the technicalities of using the app STAV, which they mostly related to age and a lack of experience. The lack of experience caused some family caregivers to disengage from the support features in STAV.” [38] “A major topic raised was privacy concerns which reduced the engagement level of some family caregivers with STAV...I do not want people to be able to Google me and come knocking on the door, maybe then start asking a lot of things or maybe pursue [the person with dementia].” [42]
**Person-centered design—future directions for improving mobile apps**		
	Prioritizing the ease of use of the apps	“All caregivers indicated that technologies targeting caregivers need to be easy to use in order to accommodate varying computer literacy...the more intuitive technologies are, the more likely they are for caregivers to adopt: When the first screen comes up, it ought to be said, ‘Am I a caregiver?’ Bam! Yes. ‘What am I looking for as a caregiver?’ Bam! And it ought to take me there. Bam! Right there. If I’m looking for my parent’s health. Bam! Take me there! Am I looking for some other help? Bam! Take me there! All of these [features], that’s great.” [46] “The participants suggested that having some hands-on practice before using the app at home would be beneficial.” [43]
	Personalization and function adaptation	“If there are four stages [of dementia], you know, breaking it down to what resources would be helpful at this stage or at this point.” [40] “Participants shared their desire for more options within the app and to have it be more personalized either to their experience or schedule...the choices were limited, and I would have liked to see more options.” [39] “A participant suggested incorporating a ‘live chat built into an app that has some resources attached to it’ and noted that this feature has the potential to build a network of people they can reach out (to).” [40]

#### Dynamic Changes in Value Perception: Complex Attitudes Toward Mobile App Adoption

##### Overview

Differences in the perceived value of mobile apps contributed to the complex attitudes among family caregivers toward their use. While most family caregivers recognized the value of mobile apps and were willing to use and recommend them to others, some exhibited insufficient perception of their value, resulting in low motivation to adopt these mobile apps. However, this value perception changed dynamically in response to the burden and progression of the disease.

##### Approving and Recommending

A total of 42% (5/12) of the studies showed positive attitudes among family caregivers toward using mobile apps. They expressed a strong need for this tool and recognized the value that it could bring, believing that it was exactly what they had been searching for a long time [30,37,41,42]. Consequently, there was an expressed desire by family caregivers to continue using mobile apps and an indication that they would recommend these apps to others [30,31].

##### Lack of Perceived Value and Preference for Alternatives

Some family caregivers felt that the functionality provided by the mobile apps failed to deliver sufficient added value, which led them to question the apps’ necessity [30,39,40,42,44]. This perspective may specifically pertain to mobile apps with limited functionality. For instance, “CLEAR Dementia Care” and “Care4Dementia” were 2 mobile apps that offered minimal functions, containing only information or tracking functions. Family caregivers noted that having these mobile apps was less beneficial than purchasing a book as they did not provide any additional information [40,44]. Family caregivers from Sweden stated that they did not use the diary and mindfulness functions because they had their preferred alternatives to use [40].

##### Adoption Attitudes That Change With Dynamic Needs

The acceptance of mobile apps among family caregivers varied according to their current caregiving burden and needs. Some family caregivers considered themselves already experienced in caregiving [42]; those with formal caregiver assistance believed that they did not need to join support programs [41], whereas family caregivers of early-stage persons with dementia felt that it was too early to seek such support [38]. Nonetheless, as the disease progressed and care demands intensified significantly, they required these mobile apps to receive more support [31,42].

#### From Tools to Partners: A Technology-Empowered Multidimensional Support System for Family Caregivers

##### Overview

The facilitation of multidimensional support functionalities enabled mobile apps to integrate into family caregivers’ lives, assisting in enhancing caregiving efficiency and capabilities, alleviating caregiving burdens, providing emotional support, fostering social connections, and promoting self-care. This caused family caregivers to shift from reacting to challenges to proactively using resources, regulating emotions, and learning continuously. Thus, mobile apps became more than “problem-solving tools”—they were now “long-term partners empowering caregiving practices.”

##### Achieved a Convenient and Efficient User Experience

A total of 67% (8/12) of the studies highlighted the mobile apps’ facility and flexibility. “The Dementia Talk” was considered visually appealing due to its multicolored layout [40], the electronic drawing platform was regarded as enjoyable [43], and the information provided in STAV was considered efficient [42]. In addition, family caregivers praised the straightforward navigation and found the technical operations to be easy and user-friendly [30,38,40,43]. More importantly, as the mobile apps were not limited by time and space, they provided greater flexibility, not only addressing physical distance constraints but also better accommodating the temporal preferences of family caregivers [31,38,39], particularly during the COVID-19 pandemic [31]. Such convenience allowed family caregivers to quickly access health care resources remotely without the need to schedule appointments or wait, thereby saving time that would otherwise be spent seeing a physician in person [38,45].

##### Facilitated the Optimization of Care Processes

In total, 75% (9/12) of the studies highlighted the positive impact of functionalities designed to optimize caregiving processes. Most mobile apps provided family caregivers with the opportunity to access educational resources and coping strategies, such as behavioral management recommendations and disease knowledge [30,31,40,42,44]. This promoted their understanding of the disease, especially for novice family caregivers. Furthermore, mobile apps with tracking or monitoring functions could serve as a central information repository, replacing traditional paperwork and assisting caregivers in storing, recording, and tracking the health status, daily activities, and medical information of the person with dementia to capture subtle changes in disease progression [38,40-42,45]. The aforementioned functions fostered proactive coping skills. Family caregivers could apply the coping strategies to translate them into specific actions for addressing unexpected real-life situations [30]. In addition, the monitoring function helped them develop daily observation habits, thereby proactively preventing various risks [45] and aiding in reviewing or reflecting on interventions after disruptive behaviors [44]. Other functions such as medication reminders [37,45], coordination of care [41], and pain assessments [37] were also considered able to increase the efficiency of the care provided.

##### Increased Social Connections and Caregivers Felt Supported

The group chat and community functions in the mobile apps held significant value as they helped family caregivers establish more social connections, alleviate feelings of loneliness, and strengthen their sense of belonging while solving problems. Through online communication with professionals, timely information exchange between family caregivers and the health team was promoted, allowing for personalized advice on addressing specific issues related to dementia, which instilled a sense of support [31,38,42,45]. STAV’s chat function enabled professionals to start group conversations, helping encourage family caregivers to communicate, especially when they were hesitant to speak up [31]. The chat function in STAV also maintained continuous professional interactions by ensuring a fixed contact person [38]. In addition, the mobile apps fostered connections between family caregivers who had similar experiences, enabling them to share caregiving challenges and experiences, thus receiving mutual support and assistance [30,31,43,46].

##### Regulated Negative Emotions

This subtheme primarily focused on the mindfulness function, an electronic painting platform, and the diary function. Family caregivers reported that mindfulness practices helped calm their minds during stressful moments, alleviating anxiety through focused breathing or meditation [31,38,39,42]. The electronic painting platform served as an effective outlet for expressing negative emotions [43]; the act of drawing not only diverted attention and allowed for a temporary escape from worries but also enabled stress relief through creative expression. The diary provided family caregivers with a private space to express their concerns, helping them reduce their psychological burdens [38,44].

##### Psychological Empowerment and Self-Care

When family caregivers used mobile apps for ≥8 weeks, some of them exhibited effects on psychological empowerment and self-care. After long-term use, the mobile app gradually transcended its role as a tool and became a source of mental stability for family caregivers. Accessing the apps made family caregivers feel reassured [38,42,45]. In addition, the mobile apps promoted more self-care. Some family caregivers using STAV realized that mobile apps could be used not only to care for persons with dementia but also to meet their own needs [38]. The mindfulness function was mentioned the most in promoting self-care. Mindfulness reminded family caregivers to actively pay attention to their own health and take time to relax while fulfilling their caregiving responsibilities. This enabled family caregivers to cultivate the ability to be aware of their state and gradually establish a present-centered mindset [31,39,42]. Meanwhile, the mindfulness function also served as a stepping stone for self-care, prompting family caregivers to proactively seek extended resources and transition from passive reliance to active daily application [39].

#### External and Internal Barriers: Challenges in Family Caregivers’ Use of Mobile Apps

##### Overview

The barriers arising from both external and internal sources collectively revealed the challenges that family caregivers face in using mobile apps. The explicit obstacles included the limited time and energy of family caregivers, physical conditions, design flaws, and device-related issues. Conversely, the implicit obstacles pertained to family caregivers’ intrinsic abilities and psychological perceptions, which influenced their choices.

##### External Environment and Technology Factors

From the perspective of family caregivers’ external environment, their health status [37,42], unforeseen life events, and a severe lack of time and energy due to caregiving responsibilities hindered the ability to consistently use mobile apps [30,31,39]. Undeniably, mobile apps’ design flaws, such as a complex interface, confusing navigation, and malfunctioning menu functions, undermined the effectiveness of immediate support [30,31,42,44]. There were also shortcomings in functional design. Family caregivers believed that some functions, such as the goal section and video functions, were unnecessary [30]. In addition, certain functions offered limited options [39,40]. For example, while family caregivers wanted to use monitoring functions to record issues, they could only select from preset options [40]. Family caregivers using STAV reported chat barriers such as limited interpersonal contact in chat rooms, single chats usually solving only 1 issue, and delayed responses causing chat termination [38]. After the intervention ended, they failed to maintain contact with health care providers through the chat function [42]. In addition, they expressed concerns about device-related issues such as compatibility, size, and costs [37,40,44].

##### Intrinsic Abilities or Psychological Factors

The limitation of mobile app use due to family caregivers’ intrinsic abilities or psychological factors was reported in 42% (5/12) of the studies. From the perspective of intrinsic abilities, a lack of technical experience posed a challenge, particularly for older family caregivers unfamiliar with technology, leading them to choose to stop using mobile apps [37,38,42]. In terms of psychological factors, emotions, the desire to focus, interest, and trust reduced family caregivers’ use of mindfulness functions [31,39]. Moreover, mobile apps’ privacy issues caused concern for family caregivers as they feared exposing their personal information online and found it difficult to discuss personal issues without compromising their privacy [31,38,42].

#### Person-Centered Design: Future Directions for Improving Mobile Apps

##### Overview

Future improvements in mobile apps should be centered on user needs. By enhancing technological adaptability and personalized design, we can create more user-friendly and efficient collaborative mobile apps, allowing them to become truly tailored assistants for individuals.

##### Prioritizing the Ease of Use of the Apps

Owing to issues such as low digital literacy and the heavy caregiving burden, family caregivers in 67% (8/12) of the studies emphasized that the ease of use of mobile apps contributed to their use. Hence, the design needs to be user-friendly, with a simple and intuitive interface, such as large fonts, a clear layout, and fewer steps to follow [31,40-42,44]. It should also incorporate multilingual support, particularly in family caregivers’ native languages, to enhance understanding [44]. In addition, using multimedia formats such as graphics and images can improve information engagement [40,42]. Providing training and guidance before use is imperative [30,31,39,40,43], such as one-on-one walk-throughs [30] and built-in video tutorials [40] for technical assistance.

##### Personalization and Function Adaptation

In total, 50% (6/12) of the studies reported family caregivers’ demand for personalized and multifunctional content in mobile apps. Regarding personalization, information about dementia care should be personalized according to the different types and stages of the disease [39,40,42,46]. At the same time, the functionalities of the mobile apps could be customized to meet different needs [39,40,46]. For those mobile apps with single or fewer functions, it would be helpful to integrate more comprehensive functions to support dementia care, such as monitoring and recording functions, reminder functions, and calendar functions [31,39,40,42,46]. Furthermore, family caregivers hoped that self-care functions such as emotional assessment, background music, and mindfulness relaxation training would be included in mobile apps because they were necessary to take care of the whole person [40,43]. Of course, mobile apps should be committed to facilitating multi-role collaboration, such as allowing family members to share caregiving information and coordinate caregiving tasks [40].

## Discussion

### Principal Findings

Our study systematically reviewed and integrated 12 qualitative studies to examine the perspectives and experiences of family caregivers of persons with dementia regarding the use of supportive mobile apps. Ultimately, 4 analytical themes were generated. We found that family caregivers’ attitudes toward using supportive mobile apps were complex and may change due to their dynamic perceptions of the mobile apps’ value. At the same time, mobile apps empowered family caregivers through multidimensional support functions, helping improve care efficiency and capacity, alleviate caregiving burdens, provide emotional support, facilitate social connections, and promote self-care. Despite significant differences in the functionalities of various mobile apps, we found that they have considerable potential to evolve from simple assistive tools into long-term partners as they can integrate into family caregivers’ daily lives and facilitate proactive changes in them. Nevertheless, external and internal barriers still affect family caregivers’ use of these apps. External factors primarily included family caregivers’ lack of time and energy, poor physical condition, design and function flaws, the devices’ memory capabilities, compatibility, and costs. Internal factors included family caregivers’ poor technological literacy, psychological resistance, and privacy concerns. In the future, more focus should be placed on person-centered improvements, especially on the ease of use of mobile apps. Mobile apps should address family caregivers' needs for personalization and multifunctionality, specifically by adapting content to disease stages and types while offering diverse support functions. Meanwhile, they should facilitate collaboration between family members and care teams to simplify caregiving tasks. According to the evidence obtained in this review, we discuss the results in this section and provide relevant recommendations for developing supportive mobile apps.

Family caregivers’ attitudes toward supportive mobile apps can be explained through the technology acceptance model [49]. This model suggests that users’ attitudes toward and intentions to use technology are driven by perceived usefulness and ease of use. Most family caregivers positively accepted and recommended mobile apps as they perceived practical value in alleviating caregiving burdens and stress. However, some family caregivers did not see mobile apps’ added value or had alternative solutions, resulting in low perceived usefulness. Their acceptance of mobile apps also varied with caregiving burdens. Family caregivers of persons with dementia in the early stages or with rich experience may not use mobile apps due to perceived limited value. However, as burdens grow, mobile apps’ advantages in professional support and self-care increase caregivers’ willingness to use them. This aligns with previous research, which highlights the crucial role of perceived usefulness in caregiver acceptance while indicating poorer acceptance among family caregivers who experience relatively low stress burdens, including caregivers in early-stage dementia care or those with substantial caregiving experience [50,51]. This dynamic change implies that mobile apps should be designed to suit family caregivers’ evolving needs as mistimed assistance may stress them [52]. Although our study did not point out family caregivers’ urgent functional needs at each stage, other research supplements this. For instance, Whitlatch and Orsulic-Jeras [53] detailed family caregivers’ needs for information, education, and sociopsychological support at different stages. Just-in-time adaptive interventions are an emerging health intervention approach that emphasizes dynamically adjusting support based on individuals’ changing states and contexts to provide help at the most needed times [54]. On the basis of this approach, the “CareQOL” app can dynamically push intervention measures according to family caregivers’ self-reported data, effectively reducing their stress [55]. Therefore, in the future, developers should explore real-time monitoring of family caregivers’ stress levels through artificial intelligence (AI) algorithms, automatically triggering matching functions to optimize mobile app design.

Findings from our study indicate that mobile apps have multidimensional functions and can provide support for family caregivers in a stressful environment. This positive impact is consistent with the stress coping model by Folkman and Lazarus [56]. Specifically, mobile apps can promote the stress adaptation of family caregivers by regulating stressors, facilitating cognitive appraisals, and providing coping processes. First, mobile apps’ user-friendly design and functions that can optimize the caregiving process help family caregivers deal with stressors such as time and space constraints and difficulties in information retrieval. This boosts their sense of stress control and coping timeliness, ultimately improving their caregiving abilities and efficiency. Second, the use of mobile apps promotes family caregivers’ cognitive restructuring of stress. Emotional regulation tools, especially mindfulness functions, allow family caregivers to cognitively reassess their emotions in the face of stress, weakening the threatening perception of stress and, thus, adjusting their responses to stress and mental states [57]. A certain period of use enables mobile apps to transcend their instrumental role and become psychological stabilizers, fostering a greater mindset of self-care. As previous research has indicated, when technology goes beyond meeting simple needs and ensures the satisfaction of psychological needs, users truly experience well-being [58]. Furthermore, due to the demands of caregiving, family caregivers often experience social isolation or difficulties in seeking help [59], and mobile apps can help build a social support network through professional collaboration and peer assistance. This not only provides dementia solutions but also enhances a sense of belonging and alleviates emotional isolation. Various support functions operate in coordination to alleviate family caregivers’ stress through different coping mechanisms, underscoring the value of developing mobile apps with comprehensive functions to foster holistic care. These findings highlight future improvement directions in mobile apps to meet family caregivers’ emotional needs and guide their self-growth. However, due to the limitations of this study, the long-term effects of mobile apps require further verification. For instance, after an intervention, family caregivers reported not maintaining contact with professionals through the chat function of the app [42]. This is in line with the results of the study by Cristancho-Lacroix et al [50]. In their online education program, almost no family caregivers continued to use the platform after the research was terminated [50]. The support provided by mobile apps may require the establishment of long-term trust as short-term impacts are often less pronounced [60]. Therefore, it is recommended that future research establish a tracking mechanism for ≥6 months or enhance user engagement through reminders, supervision, and other methods.

The barriers identified in our study have also been found in previous research [15,28,61]. Notably, many family caregivers are aged >60 years with limited technological literacy, making the use of mobile apps more challenging. The complex design of mobile apps and incompatible functions worsen their fear of technology. In addition, they are often pressed for time and, thus, have a strong need for simple, clear, and user-friendly interfaces. Furthermore, inadequate privacy measures make them cautious about mobile apps as unnecessary disclosure of sensitive information online can harm their dignity [62], and their ability to protect their online privacy appears to be lower than that of younger individuals [63]. Unfortunately, most apps developed for family caregivers of persons with dementia have very limited privacy and security measures, further hindering their use [27]. Surprisingly, our findings did not identify network barriers, likely because the included studies predominantly focused on countries with well-developed network infrastructures. However, this aspect should not be overlooked as not all mobile apps supported offline use in our included studies. Functions such as online communication and data synchronization still require internet connectivity. Rural family caregivers in another study reported a lack of internet connection as a significant barrier [19]. Optimizing the design of mobile apps and addressing a range of family caregivers’ concerns requires joint efforts from developers and policy makers. Therefore, we propose the following recommendations. Developers should adopt an age-friendly design by providing large fonts, adding images, enabling voice interactions, and supporting multiple languages. Simplifying navigation processes is essential, and this can be achieved by providing step-by-step guidance and video tutorials to reduce operational difficulties. In addition, it is necessary to fully leverage the advantages of mobile apps that support offline functionality and develop core offline functions as key components to ensure that basic services can be maintained in scenarios without network access or with unstable connectivity. From a technical security perspective, it is recommended to offer privacy setting options that allow family caregivers to determine the visibility of information and enable an anonymous mode. Furthermore, the mobile app should also safeguard data security through encryption technology and provide users with privacy protocols. Importantly, users should be involved during critical development stages such as needs analysis and testing to ensure that the design truly meets their requirements [64]. For policy makers, establishing a long-term support mechanism is crucial. On the one hand, special funds should be set up to support the development of mobile apps and related free training programs [65]. Training should include digital literacy enhancement courses and time management training courses to enhance family caregivers’ digital literacy and time management efficiency. On the other hand, technology subsidy policies should be implemented, including reimbursement of mobile app download or use fees, data flow subsidies, and other substantial support. In addition, public service platform resources should be incorporated to help create a unified service entrance in mobile apps for family caregivers, aggregating support resources such as healthcare, social assistance, legal, and financial services.

Throughout the dementia care process, the experience, capabilities, and needs of family caregivers are unique. The absence of personalized content in mobile apps may lead to ineffective care outcomes [66]. Rodriguez et al [20] developed “Brain CareNotes,” which enabled users to implement personalized settings, thereby helping users from ethnic minority groups adapt to their cultural background. The digital platform developed by Fan et al [24] integrated AI and large language models to customize and deliver the most pertinent information to users, thereby augmenting user engagement and satisfaction. This implies that a variety of tools and technologies, such as AI, customized settings, and customized questionnaires [21], can be used to assist mobile apps in achieving personalization. In addition, there are still shortcomings in the customization of functions within current mobile apps. For example, there are many general stress relief mobile apps but none specifically tailored to older family caregivers and their unique experiences and sources of stress [67]. In addition, there is a lack of integrated multifunctional mobile apps [16]. This means that family caregivers must use multiple apps to satisfy all their needs, evidently increasing their burden. Therefore, they need a comprehensive mobile app that integrates multiple functions and offers customization. Meanwhile, mobile apps should play an increased role in connecting members of the care network and promoting social service coordination. In our study, family caregivers expressed a desire for a mobile app that could provide a platform for real-time information sharing with stakeholders [40]. Previous research has developed electronic health information systems that enable information sharing among family members, community caregivers, and clinical nursing teams [68]. This sharing typically helps reduce information gaps and alleviates the stress associated with coordinating health care. However, these programs are often based on medical systems or have limited functionality [68,69] and are rarely integrated into supportive mobile apps for family caregivers of persons with dementia. Hence, this study suggests building a social network within the mobile app to improve online collaboration among primary caregivers, professionals, and other stakeholders.

### Limitations

This study has some limitations. Only English- and Chinese-language studies were included in the analysis. The exclusion of non–English- and non–Chinese-language and unpublished studies could have left out important information. The mobile apps included in the studies varied in functionality, and the family caregivers’ understanding of these functionalities also varied. Therefore, their perceptions and experiences may be biased accordingly. Moreover, none of the included studies conducted long-term interventions lasting >6 months. This resulted in a lack of sufficient evidence for us to investigate the long-term effects of mobile apps. Most of the studies (11/12, 92%) were carried out in high-income countries, so the results might not be applicable to low- and middle-income countries. Moreover, most of the included studies (9/12, 75%) lacked theoretical methodological elaboration. Thus, future research should include additional qualitative studies with clear methodologies to produce high-quality qualitative syntheses.

### Conclusions

By integrating a large amount of qualitative data, this study illustrated the complex attitudes of family caregivers of persons with dementia toward mobile apps. Through the empowerment from multidimensional supportive mobile apps, family caregivers can effectively enhance their caregiving capabilities and efficiency, alleviate caregiving burdens, reduce negative emotions, foster social connections, and promote self-focus. This continuous and multidimensional support implies that mobile apps have the potential to become trusted partners for family caregivers throughout the long-term caregiving process, assisting them in addressing care challenges at different stages. However, it is crucial to address both external and internal challenges, such as design flaws, family caregivers’ lack of technological literacy, time constraints, concerns about privacy breaches, and other device-related issues. Future improvement directions should prioritize the ease of use of technology and the development of personalized, multifunctional mobile apps, as well as promoting online collaboration among members of the care network.

## Appendix

Multimedia Appendix 1 PRISMA checklist and ENTREQ checklist.

Multimedia Appendix 2 Search strategies.

Multimedia Appendix 3 The thematic synthesis process.

## Abbreviations

AI: artificial intelligence
ENTREQ: Enhancing Transparency in Reporting the Synthesis of Qualitative Research
mHealth: mobile health
PRISMA: Preferred Reporting Items for Systematic Reviews and Meta-Analyses
