# Supplementary material for: Perspectives and Experiences of Family Caregivers Using Supportive Mobile Apps in Dementia Care: Meta-Synthesis of Qualitative Research
Source: JMIR Mhealth Uhealth. 2025 Jun 18;13:e65983. doi: 10.2196/65983 (PMC12223452; doi:10.2196/65983)
Supplement: Multimedia Appendix 2 [file mhealth_v13i1e65983_app2.docx]

**Multimedia Appendix 2. Search strategies.**

**PubMed**

Search time: 2025-3-12

| #1 | "dementia"[MeSH Terms] | 224674 |
| --- | --- | --- |
| #2 | "dement*"[Title/Abstract] OR"dementia"[Title/Abstract] OR"alzheimer*"[Title/Abstract] | 316331 |
| #3 | #1 OR #2 | 358633 |
| #4 | "caregivers"[MeSH Terms] | 56587 |
| #5 | "caregivers"[Title/Abstract] OR "carer*"[Title/Abstract] OR "caring"[Title/Abstract] OR "caretaker*"[Title/Abstract] OR "caregiver*"[Title/Abstract] OR "care giver*"[Title/Abstract] OR "spouse caregiver*"[Title/Abstract] OR "family caregiver*"[Title/Abstract] OR "informal caregiver*"[Title/Abstract] | 183537 |
| #6 | #4 OR #5 | 192679 |
| #7 | "mobile applications"[MeSH Terms] | 14386 |
| #8 | "mobile applications"[Title/Abstract] OR "APP"[Title/Abstract] OR "mobile health"[Title/Abstract] OR "mhealth"[Title/Abstract] OR "m-health"[Title/Abstract] OR "smartphone app*"[Title/Abstract] | 64968 |
| #9 | #7 OR #8 | 68775 |
| #10 | "qualitative research"[MeSH Terms] | 100619 |
| #11 | "qualitative research"[Title/Abstract] OR "qualitative study"[Title/Abstract] OR "qualitative method"[Title/Abstract] OR "narrative research"[Title/Abstract] OR "ground theory"[Title/Abstract] OR "phenomenology"[Title/Abstract] OR "descriptive"[Title/Abstract] OR "case study"[Title/Abstract] OR "focus group"[Title/Abstract] OR "interview*"[Title/Abstract] OR "investigation*"[Title/Abstract] OR "content analysis"[Title/Abstract] OR "ethnography"[Title/Abstract] OR "thematic analysis"[Title/Abstract] OR "discourse analysis"[Title/Abstract] | 1950179 |
| #12 | #10 OR #11 | 1960009 |
| #13 | #3 AND #6 AND #9 AND #12 | 109 |

**Web of Science Core Collection**

Search time: 2025-3-12

| #1 | **TS=(dementia OR dement* OR alzheimer*)** | 110347 |
| --- | --- | --- |
| #2 | **TS=(caregivers OR carer* OR caring OR caretaker* OR caregiver* OR care giver* OR spouse caregiver* OR family caregiver* OR informal caregiver*)** | 846204 |
| #3 | **TS=(APP OR mobile application OR mobile health OR mhealth OR m-health OR smartphone APP* )** | 37550 |
| #4 | **TS=(qualitative research OR qualitative study OR qualitative method OR narrative research OR ground theory OR phenomenology OR descriptive OR case study OR focus group OR interview* OR investigation* OR content analysis OR ethnography OR thematic analysis OR discourse analysis)** | 1430954 |
| #5 | **#1 AND #2 AND #3 AND #4 AND** | 154 |

| #1 | **(****dementia OR dement* OR alzheimer*):ti,ab,kw** | 26980 |
| --- | --- | --- |
| #2 | **(caregivers OR carer* OR caring, OR caretaker* OR caregiver* OR care giver* OR spouse caregiver* OR family caregiver* OR informal caregiver*):ti,ab,kw** | 343094 |
| #3 | **(APP OR mobile application OR mobile health OR mhealth OR m-health OR smartphone APP* ):ti,ab,kw** | 33034 |
| #4 | **(****qualitative research OR qualitative study OR qualitative method OR narrative research OR ground theory OR phenomenology OR descriptive OR case study OR focus group OR interview* OR investigation* OR content analysis OR ethnography OR thematic analysis OR discourse analysis):ti,ab,kw** | 359362 |
| #5 | **#1 AND #2 AND #3 AND #4 AND** | 217 |

**Cochrane Library**

Search time: 2025-3-12

**CINAHL**

Search time: 2025-3-12

| #1 | **TI ( dementia OR dement* OR alzheimer* ) OR AB ( dementia OR dement* OR alzheimer* ) OR SU ( dementia OR dement* OR alzheimer* )** | 118086 |
| --- | --- | --- |
| #2 | **TI ( caregivers OR carer* OR caring, OR caretaker* OR caregiver* OR care giver* OR spouse caregiver* OR family caregiver* OR informal caregiver* ) OR AB ( caregivers OR carer* OR caring, OR caretaker* OR caregiver* OR care giver* OR spouse caregiver* OR family caregiver* OR informal caregiver* ) OR SU ( caregivers OR carer* OR caring, OR caretaker* OR caregiver* OR care giver* OR spouse caregiver* OR family caregiver* OR informal caregiver* )** | 151705 |
| #3 | **TI ( APP OR mobile application OR mobile health OR mhealth OR m-health OR smartphone APP* ) OR AB ( APP OR mobile application OR mobile health OR mhealth OR m-health OR smartphone APP* ) OR SU ( APP OR mobile application OR mobile health OR mhealth OR m-health OR smartphone APP* )** | 30677 |
| #4 | **TI ( qualitative research OR ethnography OR thematic analysis OR discourse analysis OR qualitative study OR qualitative method OR narrative research OR phenomenology OR ground theory OR descriptive OR case study OR focus group OR interview* OR investigation* OR content analysis ) OR AB ( qualitative research OR ethnography OR thematic analysis OR discourse analysis OR qualitative study OR qualitative method OR narrative research OR phenomenology OR ground theory OR descriptive OR case study OR focus group OR interview* OR investigation* OR content analysis ) OR SU ( qualitative research OR ethnography OR thematic analysis OR discourse analysis OR qualitative study OR qualitative method OR narrative research OR phenomenology OR ground theory OR descriptive OR case study OR focus group OR interview* OR investigation* OR content analysis )** | 1601246 |
| #5 | **#1 AND #2 AND #3 AND #4** | 94 |

**PsycINFO**

Search time: 2025-3-12

| #1 | **SU (dementia OR dement* OR alzheimer*) OR TI (dementia OR dement* OR alzheimer*) OR AB (dementia OR dement* OR alzheimer*)** | 134707 |
| --- | --- | --- |
| #2 | **TI (** **caregivers OR carer* OR caring, OR caretaker* OR caregiver* OR care giver* OR spouse caregiver* OR family caregiver* OR informal caregiver* ) OR AB ( caregivers OR carer* OR caring, OR caretaker* OR caregiver* OR care giver* OR spouse caregiver* OR family caregiver* OR informal caregiver* ) OR SU ( caregivers OR carer* OR caring, OR caretaker* OR caregiver* OR care giver* OR spouse caregiver* OR family caregiver* OR informal caregiver* )** | 117279 |
| #3 | **TI ( APP OR mobile application OR mobile health OR mhealth OR m-health OR smartphone APP* ) OR AB ( APP OR mobile application OR mobile health OR mhealth OR m-health OR smartphone APP* ) OR SU ( APP OR mobile application OR mobile health OR mhealth OR m-health OR smartphone APP* )** | 19209 |
| #4 | **TI ( qualitative research OR ethnography OR thematic analysis OR discourse analysis OR qualitative study OR qualitative method OR narrative research OR phenomenology OR ground theory OR descriptive OR case study OR focus group OR interview* OR investigation* OR content analysis ) OR AB ( qualitative research OR ethnography OR thematic analysis OR discourse analysis OR qualitative study OR qualitative method OR narrative research OR phenomenology OR ground theory OR descriptive OR case study OR focus group OR interview* OR investigation* OR content analysis ) OR SU ( qualitative research OR ethnography OR thematic analysis OR discourse analysis OR qualitative study OR qualitative method OR narrative research OR phenomenology OR ground theory OR descriptive OR case study OR focus group OR interview* OR investigation* OR content analysis )** | 951096 |
| #5 | **#1 AND #2 AND #3 AND #4** | 41 |

**Embase**

Search time: 2025-3-12

| #1 | dementia:ti,ab,kw OR dement*:ti,ab,kw OR alzheimer*:ti,ab,kw | 432621 |
| --- | --- | --- |
| #2 | caregivers:ti,ab,kw OR carer*:ti,ab,kw OR caring:ti,ab,kw OR caretaker*:ti,ab,kw OR caregiver*:ti,ab,kw OR ‘care giver*’:ti,ab,kw OR ‘spouse caregiver*’:ti,ab,kw OR ‘family caregiver*’:ti,ab,kw OR ‘informal caregiver*’:ti,ab,kw | 248742 |
| #3 | APP:ti,ab,kw OR ‘mobile application’:ti,ab,kw OR ‘mobile health’:ti,ab,kw OR mhealth:ti,ab,kw OR m-health:ti,ab,kw OR ‘smartphone APP*’:ti,ab,kw | 84968 |
| #4 | ‘qualitative research’:ti,ab,kw OR ‘qualitative study’:ti,ab,kw OR ‘qualitative method’:ti,ab,kw OR ‘narrative research’:ti,ab,kw OR ‘ground theory’:ti,ab,kw OR phenomenology:ti,ab,kw OR descriptive:ti,ab,kw OR ‘case study’:ti,ab,kw OR ‘focus group’:ti,ab,kw OR interview*:ti,ab,kw OR investigation*:ti,ab,kw OR ‘content analysis’:ti,ab,kw OR ethnography:ti,ab,kw OR ‘thematic analysis’:ti,ab,kw OR ‘discourse analysis’:ti,ab,kw | 2604455 |
| #5 | **#1 AND #2 AND #3 AND #4** | 90 |

**Scopus**

Search time: 2025-3-12

| #1 | **TITLE-ABS-KEY =**"**dementia**"**OR** "**dement***" **OR**"**alzheimer***" | 479733 |
| --- | --- | --- |
| #2 | **TITLE-ABS-KEY=**"**caregivers**"**OR** "**carer***"**OR**"**caring**"**OR**"**caretaker***"**OR**"**caregiver***"**OR**"**care giver***" **OR**"**spouse caregiver***" **OR**"**family caregiver***" **OR**"**informal caregiver***" | 294780 |
| #3 | **TITLE-ABS-KEY =**"**app***"  **OR**"**application**"**OR**"**mobile application**"**OR**"**m-health**"**OR**"**mobile health**"**OR**"**smartphone app***" **OR**" **mhealth**" | 29927601 |
| #4 | **TITLE-ABS-KEY =**"qualitative research" OR "qualitative study" OR "qualitative method" OR "narrative research" OR "ground theory" OR "phenomenology" OR "descriptive" OR "case study" OR "focus group" OR "interview*" OR "investigation*" OR "content analysis" OR "ethnography" OR "thematic analysis" OR "discourse analysis" | 11821906 |
| #5 | #1 AND #2 AND #3 AND #4 AND ( LIMIT-TO ( LANGUAGE , "english" ) ) AND ( LIMIT-TO ( PUBSTAGE, "final" ) ) AND ( LIMIT-TO ( SRCTYPE , "j" ) ) | 3711 |

**CNKI**

Search time: 2025-3-12

| #1 | TKA=“痴呆 + 失智症 + 阿尔茨海默 + 认知症” | 83576 |
| --- | --- | --- |
| #2 | TKA=“照顾者 + 照护者 + 亲属 + 家庭 + 配偶 + 成年子女” | 1045634 |
| #3 | TKA=“移动应用程序 + 应用程序 + 移动应用 + 手机应用 + 微信小程序 + APP” | 12771157 |
| #4 | TKA=“质性研究 + 质性 + 现象学 + 扎根理论 + 民族志 + 人种学 + 描述性分析 + 访谈 + 案例分析 + 焦点小组” | 1111919 |
| #5 | #1 AND #2 AND #3 AND #4 | 84 |

**WanFang**

| #1 | TKA=“痴呆 OR 阿尔茨海默 OR 失智 OR 认知症” | 312384 |
| --- | --- | --- |
| #2 | TKA=“照顾者 OR 照护者 OR 亲属 OR 家庭 OR 配偶 OR 成年子女” | 1144015 |
| #3 | TKA=“移动应用程序 OR 应用程序 OR 移动应用 OR 手机应用 OR 微信小程序 OR APP” | 618799 |
| #4 | TKA=“质性研究 OR 质性 OR 现象学 OR 扎根理论 OR 民族志 OR 人种学 OR 描述性分析 OR 访谈 OR 案例分析 OR 焦点小组” | 11449674 |
| #5 | #1 AND #2 AND #3 AND #4 | 271 |

Search time: 2025-3-12

**Sinomed**

Search time: 2025-3-12

| #1 | ( "痴呆"[标题] OR "阿尔茨海默"[标题] OR "失智"[标题] OR "认知症"[标题]) OR( "痴呆"[摘要] OR "阿尔茨海默"[摘要] OR "失智"[摘要] OR "认知症"[摘要]) AND -2024[日期] | 57175 |
| --- | --- | --- |
| #2 | ( "照顾者"[标题] OR "照护者"[标题] OR "亲属"[标题] OR "家庭"[标题] OR "配偶"[标题] OR "成年子女"[标题]) OR( "照顾者"[摘要] OR "照护者"[摘要] OR "亲属"[摘要] OR "家庭"[摘要] OR "配偶"[摘要] OR "成年子女"[摘要]) AND -2024[日期] | 163951 |
| #3 | ( "移动应用程序"[标题] OR "应用程序"[标题] OR "移动应用"[标题] OR "手机应用"[标题] OR "微信小程序"[标题] OR "APP"[标题]) OR( "移动应用程序"[摘要] OR "应用程序"[摘要] OR "移动应用"[摘要] OR "手机应用"[摘要] OR "微信小程序"[摘要] OR "APP"[摘要]) AND -2024[日期] | 49661 |
| #4 | (( "质性研究"[标题] OR "质性"[标题] OR "现象学"[标题] OR "扎根理论"[标题] OR "民族志"[标题] OR "人种学"[标题] OR "描述性分析"[标题] OR "访谈"[标题] OR "案例分析"[标题] OR "焦点小组"[标题]) OR( "质性研究"[摘要] OR "质性"[摘要] OR "现象学"[摘要] OR "扎根理论"[摘要] OR "民族志"[摘要] OR "人种学"[摘要] OR "描述性分析"[摘要] OR "访谈"[摘要] OR "案例分析"[摘要] OR "焦点小组"[摘要])) AND -2024[日期] | 114001 |
| #5 | #1 AND #2 AND #3 AND #4 | 0 |

**Weipu**

Search time: 2025-3-12

| #1 | 题名或关键词=“痴呆 OR 阿尔茨海默 OR 失智 OR 认知症” | 57081 |
| --- | --- | --- |
| #2 | 题名或关键词=“照顾者 OR 照护者 OR 亲属 OR 家庭 OR 配偶 OR 成年子女” | 1007396 |
| #3 | 题名或关键词=“移动应用程序 OR 应用程序 OR 移动应用 OR 手机应用 OR 微信小程序 OR APP” | 209185 |
| #4 | 题名或关键词=“质性研究 OR 质性 OR 现象学 OR 扎根理论 OR 民族志 OR 人种学 OR 描述性分析 OR 访谈 OR 案例分析 OR 焦点小组” | 639616 |
| #5 | #1 AND #2 AND #3 AND #4 | 1 |
