# Supplementary material for: Perspectives and Experiences of Family Caregivers Using Supportive Mobile Apps in Dementia Care: Meta-Synthesis of Qualitative Research
Source: JMIR Mhealth Uhealth. 2025 Jun 18;13:e65983. doi: 10.2196/65983 (PMC12223452; doi:10.2196/65983)
Supplement: Multimedia Appendix 3 [file mhealth_v13i1e65983_app3.docx]

**Multimedia Appendix 3. The thematic synthesis process.**

| **Analytical themes** | **Descriptive themes** | **Codes** |
| --- | --- | --- |
| Dynamic changes in value perception—complex attitudes toward mobile app adoption | Approving and recommending | Recognition of the need for mobile apps |
|  |  | Future willingness to use and promotion expectations |
|  | Lack of perceived value and preference for alternatives | Lack of perceived added value of mobile apps |
|  |  | Alternative preferences |
|  | Adoption attitudes that change with dynamic  needs | Low adoption in the low-burden phase |
|  |  | Mobile app adoption linked to disease progression |
| From tools to partners—a technology-empowered  multidimensional support system for family caregivers | Achieved a convenient and efficient user experience | Positive evaluations of visual appeal and entertainment |
|  |  | Positive evaluations of ease of use |
|  |  | Unrestricted by time and space |
|  |  | Promoting the accessibility of health care |
|  |  | Advantages in special circumstances |
|  | Facilitated the optimization of care processes | Information repository |
|  |  | Tracking and monitoring |
|  |  | Other health optimization tools |
|  | Increased social connections and caregivers felt supported | Communication with professionals provides information and support |
|  |  | The benefits of chatting with professionals in promoting family caregiver communication and maintaining a fixed point of contact |
|  |  | Peer interaction promotes the sharing of experiences and emotional connections |
|  | Regulated negative emotions | Mindfulness functions promote stress relief |
|  |  | Digital painting platforms facilitate stress relief |
|  |  | Journaling promotes stress relief |
|  | Psychological empowerment and self-care | Mobile app as a source of psychological stability |
|  |  | Focus more on oneself |
|  |  | Actively seek external resources |
| External and internal barriers—challenges in family caregivers' use of mobile apps | External environment and physical conditions | Complexity of technical |
|  |  | Time and energy constraints |
|  |  | Interaction barriers |
|  |  | Redundant or limited functional design |
|  |  | Device-related factors |
|  |  | Family caregivers' physical conditions |
|  | Intrinsic abilities or psychological factors | Personal emotional resistance |
|  |  | Privacy concerns |
|  |  | Technological literacy |
| Person-centered design—future directions for improving mobile apps | Prioritizing the ease of use of the apps | Interface interaction needs |
|  |  | Multilingual needs |
|  |  | Training needs |
|  | Personalization and feature adaptation | Need for personalized information |
|  |  | Need for customized functions |
|  |  | Need for care collaboration |
